# Lentivirus-mediated knockdown of eukaryotic translation initiation factor 3 subunit D inhibits proliferation of HCT116 colon cancer cells

**DOI:** 10.1042/BSR20140078

**Published:** 2014-12-12

**Authors:** Xiaojun Yu, Bo’an Zheng, Rui Chai

**Affiliations:** *Department of Gastroenterological Surgery, Zhejiang Provincial People's Hospital, Hangzhou 310014, Zhejiang, China; †Department of Colorectal Surgery, Zhejiang Provincial People's Hospital, Hangzhou 310014, Zhejiang, China

**Keywords:** apoptosis, colon cancer, eIF3D, growth, RNA interference, AMPK, AMP-activated protein kinase, eIF, eukaryotic translation initiation factor, eIF3D, eukaryotic translation initiation factor 3 subunit D, GFP, green fluorescent protein, HEK-293T, HEK-293 cells expressing the large T-antigen of SV40 (simian virus 40), JNK, c-Jun N-terminal kinase, MPN, Mpr1-Pad1-N-terminal, MTT, 3-(4, 5-dimethylthiazol-2-yl)-2, 5-diphenyltetrazolium bromide, PARP, poly(ADP-ribose) polymerase, PCI, proteasome, COP9/signalosome, eIF3, PI, propidium iodide, PRAS40, proline-rich Akt (PKB) substrate of 40 kDa, SAPK, stress-activated protein kinase, shRNA, short hairpin RNA, TBST, Tris-buffered saline Tween-20

## Abstract

Dysregulation of protein synthesis is emerging as a major contributory factor in cancer development. eIF3D (eukaryotic translation initiation factor 3 subunit D) is one member of the eIF3 (eukaryotic translation initiation factor 3) family, which is essential for initiation of protein synthesis in eukaryotic cells. Acquaintance with eIF3D is little since it has been identified as a dispensable subunit of eIF3 complex. Recently, eIF3D was found to embed somatic mutations in human colorectal cancers, indicating its importance for tumour progression. To further probe into its action in colon cancer, we utilized lentivirus-mediated RNA interference to knock down eIF3D expression in one colon cancer cell line HCT116. Knockdown of eIF3D in HCT116 cells significantly inhibited cell proliferation and colony formation *in vitro*. Flow cytometry analysis indicated that depletion of eIF3D led to cell-cycle arrest in the G2/M phase, and induced an excess accumulation of HCT116 cells in the sub-G1 phase representing apoptotic cells. Signalling pathways responsible for cell growth and apoptosis have also been found altered after eIF3D silencing, such as AMPKα (AMP-activated protein kinase alpha), Bad, PRAS40 [proline-rich Akt (PKB) substrate of 40 kDa], SAPK (stress-activated protein kinase)/JNK (c-Jun N-terminal kinase), GSK3β and PARP [poly(ADP-ribose) polymerase]. Taken together, these findings suggest that eIF3D might play an important role in colon cancer progression.

## Abstract 2

eIF3D is essential for colon cancer cell growth, and knockdown of eIF3D resulted in a significant reduction in cell proliferation probably due to activation of AMPKα, Bad, PRAS40, SAPK/JNK and GSK3β, as well as cleavage of PARP.

## INTRODUCTION

Colon cancer, also known as colorectal cancer, rectal cancer or bowel cancer, is a cancer from uncontrolled cell growth in the colon or rectum or in the appendix. Most colon cancers originate from small, non-cancerous (benign) tumours called adenomatous polyps that form on the inner walls of the large intestine. Some of these polyps may grow into malignant colon cancers over time if they are not removed during colonoscopy. Colon cancer cells will invade and damage healthy tissue that is near the tumour causing many complications. It is commonly accepted that risk factors of colon cancer primarily include older age, male gender [1], high intake of fat, alcohol or red meat, obesity, smoking and a lack of physical exercise [2]. Approximately 10% of cases are linked to insufficient activity [3], in a summary, life style. The treatment of colorectal cancer can be aimed at curation or palliation. The decision on which aim to adopt depends on various factors, including the stage of the tumour [4]. Globally more than 1 million people get colorectal cancer every year [1] resulting in about 715 000 deaths as of 2010 up from 490 000 in 1990 [5]. As of 2008 it is the second most common cause of cancer in women and the third most common in men [6] with it being the fourth most common cause of cancer death after lung, stomach and liver cancer. It is more common in developed than developing countries [7]. There is no doubt that it is proved to be one of the most devastating tumours. Commonly used clinical treatments, including surgery, chemotherapy, radiation and palliative care have shown not effective as expected, at least to a certain extent. Genetic screening in different stages of colon cancer facilitates development of novel and personalized therapy, and this is still in process.

Regulation of protein synthesis at the level of translation initiation is fundamentally important for the control of cell proliferation under normal physiological conditions. Conversely, dysregulation of protein synthesis is emerging as a major contributory factor in cancer development. Eukaryotic protein synthesis requires the participation of eIFs (eukaryotic translation initiation factors) that mediate mRNA binding to the 40S ribosomal subunit [8, 9]. eIF3 (eukaryotic translation initiation factor 3) was one of the first initiation factors to be identified in the 1970s. The ~800 kDa mammalian eIF3 consists of 13 subunits (eIF3A–eIF3M), six of which (eIFs 3A, 3C, 3E, 3K, 3L, 3M) contain PCI (proteasome, COP9/signalosome, eIF3) domains, and two of which (eIF3F, eIF3H) contain MPN (Mpr1-Pad1-N-terminal) domains that function to promote assembly of multi-protein complexes [10,11]. These subunits are considered to be essential in complex organization because of their PCI/MPN core. The remaining subunits of eIF3 contain RRM domains (eIF3B, eIF3D, eIF3G), always draw little attention across the world since their likely dispensable role in transcription initiation. eIF3 is involved in nearly all stages of initiation, including ribosomal subunit anti-association, promoting binding of the TC to the 40S subunit, ribosomal attachment to mRNA by interacting with the eIF4G subunit of eIF4F, and scanning, and it was also shown to interact with eIF1, eIF1A, eIF2, eIF4B and eIF5 [12–14].

In keeping with the sense that transcription initiation is a vital physiological process to be regulated in eukaryotes, research into structure and ribosomal interactions of eIF3 is extensively carried out in laboratories. However, subunits, especially non-core subunits such as eIF3D, have always been away from insight of scientists. eIF3D, a subunit of eIF3 family, has been reported to inhibit HIV replication and be cleaved by HIV-1 PR, implying its role in anti-virus [15]. Moe1, the fission yeast homologue of eIF3D, was proved to be required for stable association of eIF3 [16]. Evidences have already elicited its possible non-canonical function though we could not totally exclude its role as a transcription initiation factor. Comprehensive and detailed understanding of eIF3D might need to be obtained from more in-depth research. Recently, mutation spectrum in human colorectal cancers revealed two point mutations of eIF3D in coding region, which might disrupt translation initiation, leading to tumourigenesis [17]. However, the functional role of eIF3D in colorectal cancer remains unclear. At this point, we aimed to examine the effects of eIF3D on colon cancer cell growth via lentivirus-mediated shRNA (short hairpin RNA) in one human colon cancer cell line HCT116.

## MATERIALS AND METHODS

### Cell culture

Human colon cancer cell line HCT116 and HEK-293T [HEK-293 cells expressing the large T-antigen of SV40 (simian virus 40)] were obtained from the Cell Bank of Shanghai Institute of Cell Biology, Chinese Academy of Sciences (Shanghai, China). HCT116 cells were cultured in MCCOYS’5A medium (Sigma) supplemented with 10% (v/v)FBS in 5% (v/v) CO_2_ at 37 °C. HEK293T cells were maintained in DMEM (Hyclone) supplemented with 10% FBS in 5% CO_2_ at 37 °C.

### Construction of recombinant lentivirus and gene silencing

The shRNA target sequence (5′-GCGTCATTGACATCTGC-ATGACTCGAGTCATGCAGATGTCAATGACGCTTTTTT-3′) was designed for human eIF3D gene (NM_003753.3) after screening to validate potential shRNA and inserted into the lentiviral expression vector pFH-L (Shanghai Hollybio) which contains a GFP (green fluorescent protein) gene as a reporter gene. Scrambled shRNA (5′-CTAGCCCGGTTCTCCGAACGTG-TCACGTATCTCGAGATACGTGACACGTTCGGAGAATTT-TTTTAAT-3′) was used as RNAi control. The correct insertion of the specific shRNA was verified with restriction digestion analysis and plasmid DNA sequencing. Recombinant lentiviruses were generated by triple transfection of 80% confluent HEK293T cells with modified pFH-L vector and packing plasmids pVSVG-I and pCMV□R8.92 (Shanghai Hollybio) using Lipofactinmine 2000 (Invitrogen) according to the manufacturer's instructions. Lentiviral particles (Lv-shEIF3D or Lv-shCon) were harvested at 48 h after transfection and purified by ultra-centrifugation according to previous report [18]. For lentivirus infection, HCT116 cells were seeded into six-well plates at a density of 50 000 cells per well and transduced with lentiviral particles at a MOI (multiplicity of infection) of 20. The infection efficiency was determined by counting GFP-expressing cells under fluorescence microscopy 72 h after infection.

### RNA extraction and real-time quantitative PCR

Total RNA was isolated from HCT116 cells after 5 days of infection using Trizol reagent (Gibco) according to the manufacturer's instructions. Five microgram total isolated RNA was used to synthesize the first strand of cDNA using SuperScript II RT 200 units/ml (Invitrogen). eIF3D mRNA expression was evaluated by RT-qPCR on the BioRad Connet Real-Time PCR platform with SYBR Green PCR core reagents [reaction system: 2×SYBR premix ex taq 10 μl, forward and reverse primers (2.5 μM) 0.8 μl, cDNA 5 μl, ddH_2_O (double-distilled water) 4.2 μl]. After initial denaturation at 95 °C for 1 min, a total of 40 cycles (denaturation 95 °C, 5 s; annealing extension of 60 °C, 20 s) was carried out. The absorbance value was obtained at the extension stage. Beta-actin was used as the internal reference control. The PCR primers used were as follows: eIF3D-F: 5′-CT-GGAGGAGGGCAAATACCT-3′, eIF3D-R: 5′-CTCGGTG-GAAGGACAAACTC-3′; β-actin-F: 5′-GTGGACATCCGCA-AAGAC-3′, β-actin-R: 5′-AAAGGGTGTAACGCAACTA-3′. Data were analysed using 2^−ΔΔCt^ method. Results were presented as C_T_ values, which were defined as the threshold PCR cycle number at which an amplified product is first detected. The average C_T_ was calculated for both eIF3D and β-actin, and ΔC_T_ was determined as the ratio of the mean of the triplicate C_T_ values for eIF3D to the mean of the triplicate C_T_ values for β-actin. Each experiment was performed in triplicate and repeated three times.

### Western blotting

After infection for 5 days, HCT116 cells were washed with ice-cold PBS and then lysed in 2× SDS sample buffer [100 mM Tris–HCl (pH 6.8), 10 mM EDTA, 4% (w/v) SDS, 10% (v/v) glycine] for 1 h at 4 °C. The lysates were clarified by centrifugation at 13 000× ***g*** for 30 min at 4 °C and the supernatants were employed for further analysis. The total protein concentration was estimated using BCA (bicinchoninic acid) protein assay kit. Protein samples (30 μg) were loaded and electrophoresed in a SDS–PAGE (10% gel) at 50 V for 3 h, and subsequently transferred to PVDF membranes (Millipore) at 300 mA for 1.5 h. After being blocked with TBST (Tris-buffered saline Tween-20) [20 mM Tris (pH7.6), 150 mM NaCl, 0.01% Tween-20] containing 5% (w/v) non-fat dried skimmed milk powder for 1 h at room temperature, membranes were probed with rabbit anti-EIF3D (abcam, #ab155419, dilution 1:1000), or mouse anti-GAPDH (Santa cruz, #Sc-32233, dilution 1:60 000) overnight at 4 °C. After washing by TBST, the membrane was incubated with HRP (horseradish peroxidase)-labelled anti-rabbit (Santa cruz, #Sc-2054, dilution 1:5000) or anti-mouse (Santa cruz, #Sc-2005, dilution 1:5000) secondary antibody at room temperature for 2 h. The membranes were analysed using super ECL detection reagent. Each experiment was repeated three times.

### MTT [3-(4, 5-dimethylthiazol-2-yl)-2, 5-diphenyltetrazolium bromide] cell viability assay

Briefly, HCT116 cells from different groups (Lv-shEIF3D, Lv-shCon, Con) were seeded in 96-well plates at a density of 2000 cells per well. At indicated time points, MTT was added into each well at a final concentration of 5 mg/ml for 4 h. Acidic isopropanol [10% SDS, 5% (v/v) isopropanol and 0.01 mol/l HCl] was then added to stop the reaction and measured with an ELISA reader (Bio-Rad) at a wavelength of 595 nm. Viability of cells was calculated from theoretical absorbance. Each experiment was performed in triplicate and repeated three times.

### Colony formation assay

In order to assay monolayer colony formation, stably transduced HCT116 cells from different groups (Lv-shEIF3D, Lv-shCon, Con) after 96 h infection were plated into six-well plates at a density of 400 cells per well. After culture for 7 days, cells were fixed with paraformaldehyde and then stained with crystals purple as described in previous report [19]. The number of colonies was counted. Each experiment was performed in triplicate and repeated three times.

### Cell-cycle analysis

Cell-cycle progression was determined by PI (propidium iodide) staining using a flow cytometer (FACS Calibur, BD Biosciences). Briefly, after 3 days of infection at an MOI of 20, HCT116 cells were reseeded in 6-cm dishes (200 000 cells per dish) and cultured for 40 h at 37 °C. After trypsinization treatment, HCT116 cells in each well were harvested at a cell confluence of 80%, followed by washing with ice-cold PBS and fixing with 70% (v/v) cold alcohol. After centrifugation, cells were resuspended in PI/RNase/PBS (100 μg/ml propidium iodide and 10 μg/ml RNase A) solution and incubated in dark for 30 min at room temperature. The suspension was filtered through a 50-mm nylon mesh, and the DNA content of stained nuclei was analysed by PI staining. PI uptake was analysed by fluorescence activated cell sorting on flow cytometer. Each experiment was performed in triplicate and repeated three times.

### Intracellular signalling assay

Phosphorylation and proteolysis are two widespread covalent post-translational modifications that represent important regulatory mechanisms in biology. Detection of these modifications on a set of cellular proteins playing a well-understood role in cell biology can provide a broad snapshot of intracellular signalling. PathScan® intracellular signalling array kit (Cell signaling technology, #7323) was taken to detect alteration of signalling molecules. HCT116 cell lysate was prepared as mentioned previously. Detection procedure was performed according to the protocol provided by CST. Each experiment was repeated three times.

### Statistical analysis

All statistical analyses were performed using SPSS13.0 software. The differences between groups were compared using Student's *t* test, and data were expressed as means±S.D. of three independent experiments. Statistically significant difference was accepted at *P*<0.05.

## RESULTS

### Lentivirus-mediated RNAi effectively decreases eIF3D expression in HCT116 cells

**Figure 1:**
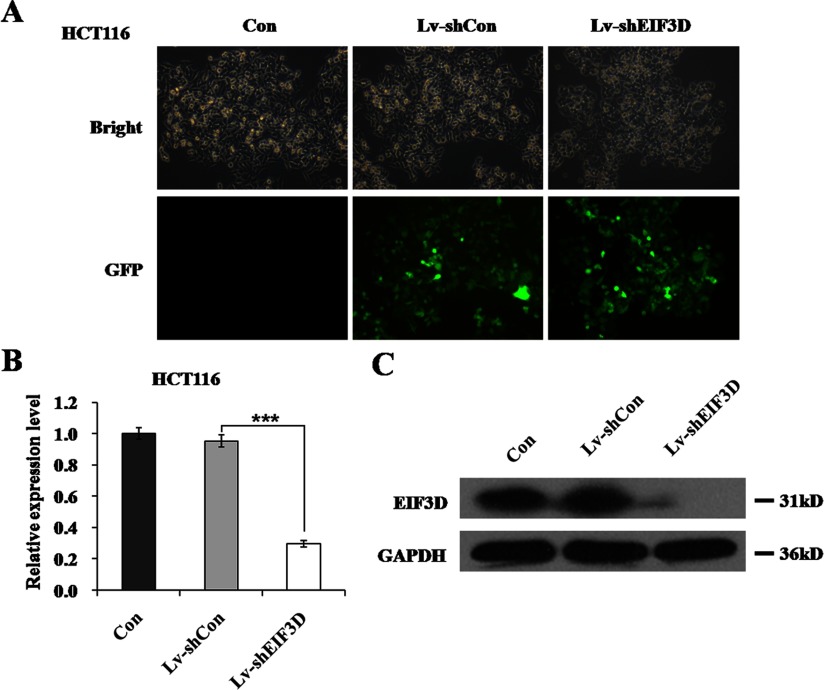
Knockdown of eIF3D through lentivirus-mediated RNAi (**A**) Representative graphs of HCT116 cells infected with indicated lentivirus at MOI of 20 were shown. Efficiency of lentivirus infection was reflected from green fluorescence of GFP. (**B**) eIF3D mRNA levels were measured by RT–qPCR in three groups of HCT116 cells after 5 days infection. β-actin was used as an internal gene. (**C**) eIF3D protein levels were detected by western blot analysis in three groups of HCT116 cells after 5 days infection. GAPDH was used as loading control. ***P<0.001, compared with Lv-shCon.

eIF3D, which is of our interest, has been reported to mutate at two points in human colorectal cancer identified by mutation spectrum [17]. We sought to determine its functional role through lentivirus-mediated RNAi in colon cancer cell line HCT116. As shown in Figure 1(A), the infection efficiency was visibly high (>80%) as evidenced by reporter gene GFP expression. Knockdown efficiency was evaluated by RT–qPCR (quantitative PCR) and Western blotting. As shown in Figure 1(B), endogenous eIF3D mRNA was significantly reduced in the Lv-shEIF3D group with 68.9% of inhibition rate, compared with the Lv-shCon group (*P*<0.001). Western blotting analysis further confirmed the silencing of eIF3D as its protein level was also down-regulated in HCT116 cells following Lv-shEIF3D treatment (Figure 1C). Therefore we could conclude that lentivirus-mediated knockdown of eIF3D successfully inhibited its endogenous expression level in HCT116 cells.

### Down-regulation of eIF3D alleviates cell proliferation in HCT116 cells

**Figure 2:**
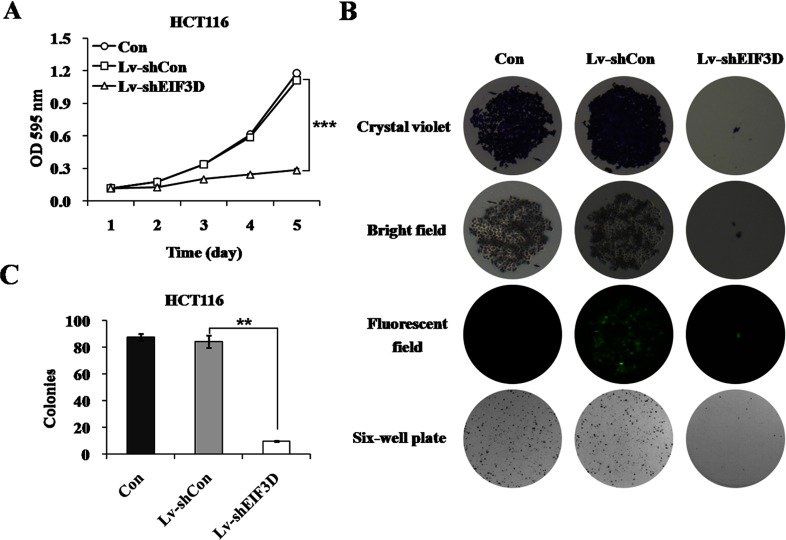
Effects of eIF3D knockdown on HCT116 cell proliferation and colony formation (**A**) The monolayer growth rates of HCT116 cells from three groups were determined by MTT assay. Statistical plots were made according to the results from three independent experiments. ***P<0.001, compared with Lv-shCon. (**B**) Down-regulation of eIF3D inhibited colony formation ability. Representative photographs of single colony and total colonies in plates were shown. (**C**) The average number of colonies in each plate was shown in the histogram. **P<0.01, compared with Lv-shCon.

Based on the efficient knockdown, we examined whether the down-regulation of eIF3D influenced the malignant phenotype of HCT116 cells, tending to elucidate its possible role in colon cancer. Cell proliferation was firstly examined in HCT116 cells following Lv-shEIF3D treatment using an MTT assay. As shown in Figure 2(A), the proliferative rate of EIF3D knockdown cells was significantly decreased compared with the Lv-shCon group after culture for 4 days (0.245±0.005 versus 0.587±0.006, *P*<0.001) or 5 days (0.284±0.013 versus 1.111±0.026, *P*<0.001), while there was no significant difference between Lv-shCon and Con groups, indicating the efficient inhibition of cell proliferation in Lv-shEIF3D infected HCT116 cells. Furthermore, the effect of eIF3D knockdown on *in vitro* tumourigenicity of HCT116 cells was tested by colony formation assay. As shown in Figure 2(B), Lv-shEIF3D obviously reduced the size of single colony and the number of colonies formed in HCT116 cells. The colonies number in the Lv-shEIF3D group was apparently fewer than in the Lv-shCon group (9.7±0.6 versus 84.0±4.6, *P*<0.01; Figure 2C). These results strongly suggest that eIF3D is indispensable for HCT116 cell proliferation.

### Down-regulation of eIF3D induces cell-cycle arrest and apoptosis in HCT116 cells

**Figure 3:**
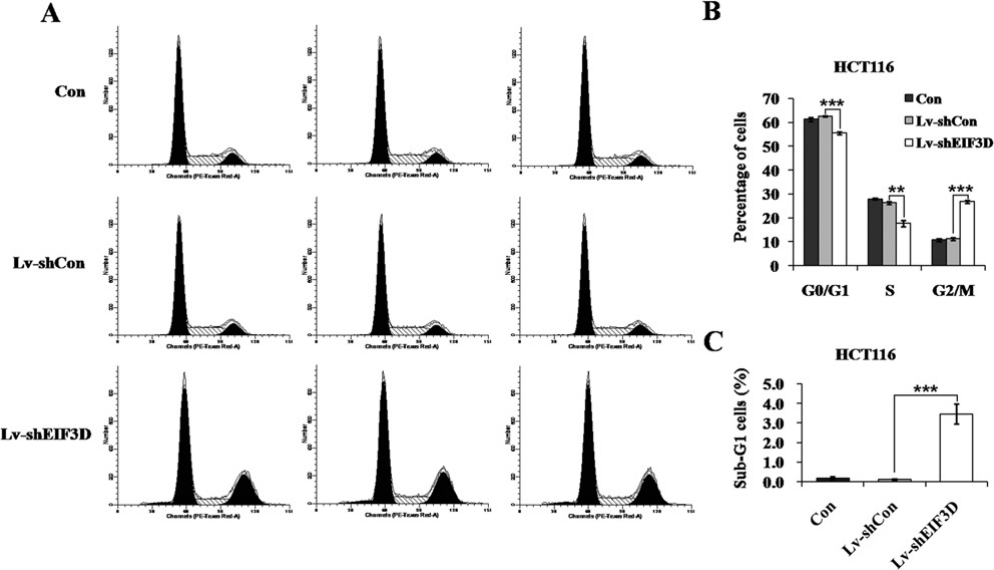
Down-regulation of eIF3D induces cell-cycle arrest as well as apoptosis (**A**) Cell-cycle distribution of HCT116 cells was analysed by FACS. (**B**) The percentage of HCT116 cells in G2/M phase was significantly higher in Lv-shEIF3D group than that in control groups. (**C**) The percentage of HCT116 cells in sub-G1 phase was significantly higher in Lv-shEIF3D group than that in control groups. **P<0.01, ***P<0.001, compared with Lv-shCon.

As extensively accepted, cancer cells always exert characteristic uncontrolled cell cycle. Many clinical used drugs for cancer therapy specially targeted to cell-cycle arrest, and this was considered as a promising lead to cancer therapeutics. Given that the dysregulation of cell-cycle progression is highly related with the abnormal cell proliferation, we investigated the cell-cycle progression to further explore the mechanisms underlying the inhibition of cell growth by eIF3D knockdown. The cell-cycle distribution of HCT116 cells after eIF3D knockdown was analysed through FACS assay (Figure 3A). We found that eIF3D knockdown cells were blocked in the G2/M phase, compared with control cells. The cell percentage in the G2/M phase was markedly increased from 11.22±0.67% in the Lv-shCon group to 26.86±0.63% in the Lv-shEIF3D group (*P*<0.001). The cell populations of G_0_/G_1_ phase and S phase were concomitantly decreased following Lv-shEIF3D treatment (Figure 3B). Moreover, knockdown of eIF3D in HCT116 cells led to an excess accumulation in the sub-G1 phase representing apoptotic cells (Figure 3C). These results suggest that knockdown of eIF3D inhibited colon cancer cell growth possibly by inducing cell-cycle arrest as well as apoptosis.

### Modifications of effector proteins were altered in eIF3D knockdown HCT116 cells

**Figure 4:**
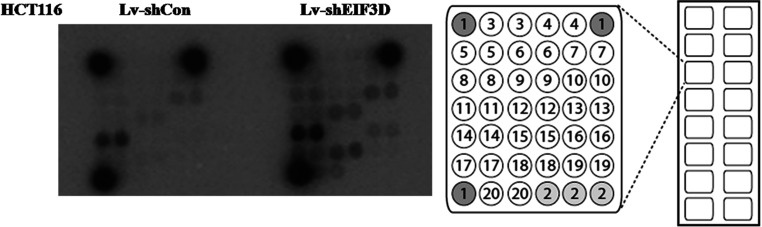
Alterations of protein modification are detected through intracellular signalling assay Modifications of effector proteins were analysed in HCT116 cells from two groups. The data could be directly read out from the spot intensity showed in the left graph. The distribution of 20 protein modifications in one chip was shown in the right graph.

To explore the underlying signalling pathways mediated by eIF3D in colon cancer cells, PathScan® intracellular signalling array kit was utilized. The detected signalling pathways were involved in cell proliferation, growth, cell cycle, survival or apoptosis, including MAPK/ERK cascade, p38 and JNK(c-Jun N-terminal kinase) MAPKs, Stat1 and Stat3, Akt [PKB (protein kinase B)], mTOR (mammalian target of rapamycin), AMPK (AMP-activated protein kinase), HSP (heat-shock protein) 27, p53 and caspase-3. The alterations of modifications occurred in signalling proteins were shown in Figure 4. To our surprise, down-regulation of eIF3D in HCT116 cells resulted in a series of phosphorylation upregulation, including AMPKα (Thr^172^), Bad (Ser^112^), PRAS40 (proline-rich Akt substrate of 40 kDa; Thr^246^), SAPK (stress-activated protein kinase)/JNK (Thr^183^/Tyr^185^), GSK3β (Ser^9^), as well as the cleavage of PARP [poly(ADP-ribose) polymerase] (Table 1). These data suggest that the involvement of eIF3D in colon cancer cell growth may be partly via the modulation of related signalling proteins.

**Table 1:** Representative modification from different wells in one chip

Target	Phosphorylation	Site	Modification	Lv-shEIF3D versus Lv-shCon
8	AMPKα	Thr^172^	Phosphorylation	Up-regulation
12	Bad	Ser^112^	Phosphorylation	Up-regulation
14	PRAS40	Thr^246^	Phosphorylation	Up-regulation
17	SAPK/JNK	Thr^183^/Tyr^185^	Phosphorylation	Up-regulation
18	PARP	Asp^214^	Cleavage	Up-regulation
20	GSK-3β	Ser^9^	Phosphorylation	Up-regulation

## DISCUSSION

eIF3D has commonly been described as a subunit of eIF3 complex, participating in nearly all stages of transcription initiation. However, acquaintance with eIF3D is little in detail since it was identified as a dispensable unit of eIF3 complex for decades. Recently, some studies have found that eIF3D is associated with malignant mesothelioma and gastric cancer, implying its aberrant expression may participate in many pathological processes [20]. eIF3D is ubiquitously expressed in colon cells. Two point mutations of eIF3D were reported by mutation spectrum in human colorectal cancers [17]. Therefore in the present study, we aimed to explore the functional role of eIF3D in colon cancer. Lentivirus-loaded shRNA targeting for eIF3D was constructed to knock down its expression in colon cancer cell line HCT116. MTT and colony formation assay suggested that eIF3D was indispensable for cell proliferation. FACS assay further indicated that knockdown of eIF3D resulted in cell-cycle arrest in the G2/M phase, especially in the sub-G1 phase, which represents apoptotic cells [21], which revealed its key role in cell-cycle control and apoptosis.

AMPK is an energy sensor that is activated by phosphorylation at Thr^172^ in response to elevated AMP levels. AMPK acts as a metabolic master switch regulating several intracellular systems fatty acid metabolism, protein synthesis and cell growth [22–24]. Phosphorylation of the pro-apoptotic protein Bad inhibits cell proliferation and promotes apoptosis while inhibition of it increases cell growth rate [25]. Previous studies showed that PRAS40 knockdown reduced the ability of tumour TNF (necrosis factor—α) and cycloheximide induced apoptosis in HeLa cells, indicating the pro-apoptotic function of PRAS40 [26]. Phosphorylation of SAPK/JNK at Thr^183^ and Tyr^185^ reflects its activation in response to stressful conditions, genotoxicity or anticancer drugs [27,28]. Phosphorylation of the multifunctional kinase GSK-3β at Ser^9^ [29,30] inhibits its activity. Inhibition of GSK-3β enhances reovirus-induced apoptosis in colon cancer cells [31]. The presence of cleaved PARP is one of the most used diagnostic tools for the detection of apoptosis in many cell types [32]. In the present study, phosphorylation of AMPKα, Bad, PRAS40, SAPK/JNK and GSK3β, as well as cleavage of PARP was elevated in eIF3D knockdown group, adding the evidence that depletion of eIF3D could suppress colon cancer cell growth via the induction of cell-cycle arrest and apoptosis. Furthermore, comprehensive acquaintance with these signalling pathways still needs to be obtained. Our data were supported by a recent study showing that knockdown of eIF3D suppressed mesothelioma cell proliferation and increased apoptosis, identifying eIF3D as a potential drug target in malignant mesothelioma [33]. eIF3D was also identified among a group of proteins, responsible for protein synthesis, that made up an acquired resistance signature in gastric cancer patients, suggesting that eIF3D was up-regulated and may bear predictive value in patients establishing resistance against a combined treatment of cisplatin and flurouracil [34]. Up to now, we could not conclude whether eIF3D functioned as a transcription initiator. These results have already provided possible clues of eIF3D multifarious effect.

In recent years, many eIFs have been found to influence cancer development and progression, representing future drug targets in clinical oncology [20]. Antisense therapy has arisen as a promising therapy for many diseases including cancer. When the genetic sequence of a particular gene is known to be causative of a particular disease, it is possible to synthesize a strand of nucleic acid that will bind to the mRNA (messenger RNA) produced by that gene and inactivate it, effectively turning that gene ‘off’. This is because mRNA has to be single stranded for it to be translated. Alternatively, the strand might be targeted to bind a splicing site on pre-mRNA and modify the exon content of an mRNA [35]. As of 2012, some 40 antisense oligonucleotides and siRNAs (small interfering RNAs) were in clinical trials, including over 20 in advanced clinical trials (Phase II or III) [36,37]. If eIF3D is proved to be the pivotal factor in colon cancer development and progression, then antisense therapy targeting eIF3D will be intriguing.

## References

[1] Cunningham D., Atkin W., Lenz H. J., Lynch H. T., Minsky B., Nordlinger B., Starling N. (2010). Colorectal cancer. Lancet.

[2] Watson A. J., Collins P. D. (2011). Colon cancer: a civilization disorder. Dig. Dis..

[3] Lee I. M., Shiroma E. J., Lobelo F., Puska P., Blair S. N., Katzmarzyk P. T., Lancet Physical Activity Series Working G. (2012). Effect of physical inactivity on major non-communicable diseases worldwide: an analysis of burden of disease and life expectancy. Lancet.

[4] Stein A., Atanackovic D., Bokemeyer C. (2011). Current standards and new trends in the primary treatment of colorectal cancer. Eur. J. Cancer.

[5] Lozano R., Naghavi M., Foreman K., Lim S., Shibuya K., Aboyans V., Abraham J., Adair T., Aggarwal R., Ahn S. Y. (2012). Global and regional mortality from 235 causes of death for 20 age groups in 1990 and 2010: a systematic analysis for the global burden of disease study 2010. Lancet.

[6] Gansler T., Ganz P. A., Grant M., Greene F. L., Johnstone P., Mahoney M., Newman L. A., Oh W. K., Thomas C. R., Thun M. J. (2010). Sixty years of CA: a cancer journal for clinicians. CA: Cancer J. Clin..

[7] Merika E., Saif M. W., Katz A., Syrigos K., Morse M. (2010). Review. Colon cancer vaccines: an update. In Vivo.

[8] Pestova T. V., Kolupaeva V. G. (2002). The roles of individual eukaryotic translation initiation factors in ribosomal scanning and initiation codon selection. Genes Dev..

[9] Algire M. A., Maag D., Lorsch J. R. (2005). Pi release from eIF2, not GTP hydrolysis, is the step controlled by start-site selection during eukaryotic translation initiation. Mol. Cell.

[10] Pena V., Liu S., Bujnicki J. M., Luhrmann R., Wahl M. C. (2007). Structure of a multipartite protein–protein interaction domain in splicing factor prp8 and its link to retinitis pigmentosa. Mol. Cell.

[11] Enchev R. I., Schreiber A., Beuron F., Morris E. P. (2010). Structural insights into the COP9 signalosome and its common architecture with the 26S proteasome lid and eIF3. Structure.

[12] Hinnebusch A. G. (2006). eIF3: a versatile scaffold for translation initiation complexes. Trends Biochem. Sci..

[13] Jackson R. J., Hellen C. U., Pestova T. V. (2010). The mechanism of eukaryotic translation initiation and principles of its regulation. Nat. Rev. Mol. Cell Biol..

[14] Valasek L. S. (2012). ‘Ribozoomin’-translation initiation from the perspective of the ribosome-bound eukaryotic initiation factors (eIFs). Curr. Protein Pept. Sci..

[15] Jager S., Cimermancic P., Gulbahce N., Johnson J. R., McGovern K. E., Clarke S. C., Shales M., Mercenne G., Pache L., Li K. (2011). Global landscape of HIV-human protein complexes. Nature.

[16] Bandyopadhyay A., Lakshmanan V., Matsumoto T., Chang E. C., Maitra U. (2002). Moe1 and spInt6, the fission yeast homologues of mammalian translation initiation factor 3 subunits p66 (eIF3d) and p48 (eIF3e), respectively, are required for stable association of eIF3 subunits. J. Biol. Chem..

[17] Yin H., Liang Y., Yan Z., Liu B., Su Q. (2013). Mutation spectrum in human colorectal cancers and potential functional relevance. BMC Med. Genet..

[18] Sakoda T., Kasahara N., Hamamori Y., Kedes L. (1999). A high-titer lentiviral production system mediates efficient transduction of differentiated cells including beating cardiac myocytes. J. Mol. Cell. Cardiol..

[19] Xu M., Wang Y., Chen L., Pan B., Chen F., Fang Y., Yu Z., Chen G. (2014). Down-regulation of ribosomal protein S15A mRNA with a short hairpin RNA inhibits human hepatic cancer cell growth *in vitro*. Gene.

[20] Spilka R., Ernst C., Mehta A. K., Haybaeck J. (2013). Eukaryotic translation initiation factors in cancer development and progression. Cancer Lett..

[21] Riccardi C., Nicoletti I. (2006). Analysis of apoptosis by propidium iodide staining and flow cytometry. Nat. Protoc..

[22] Carling D., Mayer F. V., Sanders M. J., Gamblin S. J. (2011). AMP-activated protein kinase: nature's energy sensor. Nat. Chem. Biol..

[23] Mihaylova M. M., Shaw R. J. (2011). The AMPK signalling pathway coordinates cell growth, autophagy and metabolism. Nat. Cell Biol..

[24] Hardie D. G., Ross F. A., Hawley S. A. (2012). AMPK: a nutrient and energy sensor that maintains energy homeostasis. Nat. Rev.: Mol. Cell Biol..

[25] Konishi Y., Lehtinen M., Donovan N., Bonni A. (2002). Cdc2 phosphorylation of BAD links the cell cycle to the cell death machinery. Mol. Cell.

[26] Thedieck K., Polak P., Kim M. L., Molle K. D., Cohen A., Jeno P., Arrieumerlou C., Hall M. N. (2007). PRAS40 and PRR5-like protein are new mTOR interactors that regulate apoptosis. PloS ONE.

[27] Satomi Y. (2012). Fucoxanthin induces GADD45A expression and G1 arrest with SAPK/JNK activation in LNCap human prostate cancer cells. Anticancer Res..

[28] Chang L., Karin M. (2001). Mammalian MAP kinase signalling cascades. Nature.

[29] Cross D. A., Alessi D. R., Cohen P., Andjelkovich M., Hemmings B. A. (1995). Inhibition of glycogen synthase kinase-3 by insulin mediated by protein kinase B. Nature.

[30] Jacobs K. M., Bhave S. R., Ferraro D. J., Jaboin J. J., Hallahan D. E., Thotala D. (2012). GSK-3beta: A bifunctional role in cell death pathways. Int. J. Cell Biol..

[31] Min H. J., Koh S. S., Cho I. R., Srisuttee R., Park E. H., Jhun B. H., Kim Y. G., Oh S., Kwak J. E., Johnston R. N., Chung Y. H. (2009). Inhibition of GSK-3beta enhances reovirus-induced apoptosis in colon cancer cells. Int. J. Oncol..

[32] Bressenot A., Marchal S., Bezdetnaya L., Garrier J., Guillemin F., Plenat F. (2009). Assessment of apoptosis by immunohistochemistry to active caspase-3, active caspase-7, or cleaved PARP in monolayer cells and spheroid and subcutaneous xenografts of human carcinoma. J. Histochem. Cytochem..

[33] Sudo H., Tsuji A. B., Sugyo A., Kohda M., Sogawa C., Yoshida C., Harada Y. N., Hino O., Saga T. (2010). Knockdown of COPA, identified by loss-of-function screen, induces apoptosis and suppresses tumor growth in mesothelioma mouse model. Genomics.

[34] Kim H. K., Choi I. J., Kim C. G., Kim H. S., Oshima A., Michalowski A., Green J. E. (2011). A gene expression signature of acquired chemoresistance to cisplatin and fluorouracil combination chemotherapy in gastric cancer patients. PloS ONE.

[35] Morcos P. A. (2007). Achieving targeted and quantifiable alteration of mRNA splicing with Morpholino oligos. Biochem. Biophys. Res. Commun..

[36] Bennett C. F., Swayze E. E. (2010). RNA targeting therapeutics: molecular mechanisms of antisense oligonucleotides as a therapeutic platform. Annu. Rev. Pharmacol. Toxicol..

[37] Watts J. K., Corey D. R. (2012). Silencing disease genes in the laboratory and the clinic. J. Pathol..

